# “I decide myself”- A qualitative exploration of end of life decision making processes of patients and caregivers through Advance Care Planning

**DOI:** 10.1371/journal.pone.0252598

**Published:** 2021-06-18

**Authors:** Priya Lall, Oindrila Dutta, Woan Shin Tan, Paul Victor Patinadan, Natalie Q. Y. Kang, Chan Kee Low, Josip Car, Andy Hau Yan Ho

**Affiliations:** 1 Lincoln International Institute for Rural Health, College of Social Science, University of Lincoln, Lincoln, United Kingdom; 2 London Interdisciplinary School, London, United Kingdom; 3 Psychology Programme, School of Social Sciences, Nanyang Technological University, Singapore, Singapore; 4 NTU Institute for Health Technologies, Interdisciplinary Graduate School, Nanyang Technological University, Singapore, Singapore; 5 Health Services and Outcomes Research Department, National Healthcare Group, Singapore, Singapore; 6 Economics Programme, School of Social Sciences, Nanyang Technological University, Singapore, Singapore; 7 Centre of Population Health Sciences, Lee Kong Chian School of Medicine, Nanyang Technological University, Singapore, Singapore; 8 Global eHealth Unit, Department of Primary Care and Public Health, School of Public Health, Imperial College London, London, United Kingdom; 9 Palliative Care Centre for Excellence in Research and Education, Singapore, Singapore; Universidade Estadual Paulista Julio de Mesquita Filho Faculdade de Medicina Campus de Botucatu, BRAZIL

## Abstract

**Background:**

The Singapore national Advance Care Planning (ACP) programme was launched in 2011 with the purpose of ensuring that healthcare professionals are fully aware of patients’ treatment preferences. There is little research assessing the performance of such programmes in ethnically diverse Asian countries; hence, the purpose of this study was to qualitatively examine patients and caregivers’ experiences with the ACP programme.

**Method:**

We conducted interviews with 28 participants, thirteen of whom identified as proxy decision makers (PDMs) and the remainder as patients. Interviews focused on respondents’ experiences of chronic illness and of participating in the ACP programme. Textual data was analysed through a framework analysis approach.

**Results:**

Participants’ narratives focused on four major themes with 12 subthemes: a) Engagement with Death, factors influencing respondents’ acceptance of ACP; b) Formation of Preferences, the set of concerns influencing respondents’ choice of care; c) Choice of PDM, considerations shaping respondents’ choice of nominated health spokesperson; and d) Legacy Solidification, how ACP is used to ensure the welfare of the family after the patient passes. These findings led to our development of the directive decision-making process framework, which delineates personal and sociocultural factors influencing participants’ decision-making processes. Respondents’ continual participation in the intervention were driven by their personal belief system that acted as a lens through which they interpreted religious doctrine and socio-cultural norms according to their particular needs.

**Conclusion:**

The directive decision-making process framework indicated that ACP could be appropriate for the Asian context because participants displayed an awareness of the need for ACP and were able to develop a concrete treatment plan. Patients in this study made decisions based on their perceived long-term legacy for their family, who they hoped to provide with a solid financial and psychological foundation after their death.

## Background

Currently the global population is undergoing an epidemiologic transition as individuals’ lifespans increase due to technological and medical advancement [1], which has resulted in the percentage of people aged 60 years or older being projected to rise from 11% in 2000 to 22% in 2050 on a global level [2]. This demographic transition could particularly impact Asia [3], which already contains an estimated third of the global elderly population and encompasses countries with rapidly ageing populations, such as Singapore, China and Japan [4, 5]. Within these countries, it is expected that demand for health and social services, including palliative care, may swell as more individuals experience degenerative illnesses associated with ageing, e.g. dementia.

Although there is an increasing need for palliative care in Asian countries, there remains a lack of adequate provision of such services in many of them [6]. Even in nations that reportedly have accessible palliative care services, such as Singapore, individuals End-of-Life (EoL) care preferences are rarely realised. A survey on Singaporeans’ attitudes towards death in 2014 revealed that around three quarters of participants preferred to die at home, although around a quarter of deaths take place in these settings [7]. This particular trend could be driven by healthcare professionals (HCPs) and caregivers; lack of knowledge of patients’ EoL care preferences [8], which can result in them undergoing painful, technologically-intensive and unnecessary treatments.

The ‘Living Matters’ Advance Care Planning (ACP) programme was launched at the national level in Singapore in acute care settings in 2011, with the purpose of ensuring that HCP and carers were aware of patients’ treatment preferences [9, 10]. This programme was based on the ‘Respecting Choices’ model [11, 12], which has an underlying philosophy that patients’ could only fully attain autonomy over their EoL care by articulating a treatment plan in accordance with their personal goals [13]. The ‘Living Matters’ programme develops a treatment plan in a similar fashion to that of the ‘Respecting Choices’ model, wherein participants are encouraged to articulate their treatment preferences through a series of structured discussions with their HCPs and carers focusing on their personal values and beliefs. Resulting preferences are usually documented and endorsed by the responsible physicians in order to guide future HCP involved in care of the patient towards the end of their lives. Family carers attending the ACP conversations are expected to later act as proxy decision makers (PDMs), who may communicate the patients’ goals and treatment preferences should they be too incapacitated to do so, or when the ACP discussion has not been documented.

By 2017, over 2000 HCP were trained to conduct ACP conversations [9], resulting in an estimated 10,000 ACP discussions being completed in Singapore. Although uptake of ACP discussions remains relatively low, a recent evaluation of the ‘Living Matters’ programme indicates that it may improve quality of EoL care by ensuring that patients’ treatment preferences are adhered to [14]. Moreover, findings from evaluative studies conducted mostly in Western countries suggest that ACP has the potential to benefit both patients [15–18] and caregivers [19, 20]. These studies indicate that ACP enhanced patients’ sense of autonomy [21] and satisfaction with care [18, 20] as it offered HCPs an opportunity to empathetically listen to them [17]. Meanwhile, caregivers displayed improved psychosocial outcomes after the patients’ death in comparison to those who had not undertaken the intervention [22].

It is possible that participation in ACP discussions has a negative impact on dynamics within families [23]. In one study conducted in Denmark, participation sometimes obstructed communication on EoL care because parties involved were overwhelmed or offended by the subject matter [24]. This negative outcome could potentially be amplified within a Southeast Asian context owing to filial piety [25, 26] and pervasive death taboos, which can result in fears that discussion of death could potentially hasten a patient’s demise [27].

Despite these concerns, there remains relatively little research on patients’ and caregivers’ perspectives on ACP in South-East Asian countries with population compositions like that of Singapore [28], which is mostly ethnically Chinese [25]. Research on the performance of ACP and/or Advance Directives in South-East Asian countries revealed that patients’ receptivity and decision-making processes were markedly different to that of white patients living in Western countries [28]. In contrast to these patients, whose interest in ACP were driven by a desire for autonomy, ethnically Chinese patients’ receptivity were influenced by their families’ acceptance of the intervention [29]. As such, they tended to collectively make decisions on their EoL care in concert with family members, in some cases deferring their decisions on treatment to their physicians.

Given that ethnically Chinese patients’ decision-making processes may contrast to that originally envisioned in the ‘Respecting Choices’ model, which emphasises patient autonomy within its underlying philosophy, this paper seeks to examine patients and PDMs experiences of ACP in Singapore. As such, it encompasses factors influencing patients’ receptivity to the programme and the decision-making processes they undertook when choosing their EoL care preferences and a PDM.

## Methods

This paper draws on data generated from the qualitative component of a largescale national level mixed methods evaluation of the ACP programme in Singapore. The qualitative component was spearheaded by AH, who has extensive experience conducting research on EoL care in Asian countries. It used many forms of data collection, including focus group discussions with HCPs, individual interviews with patients and their designated health spokesperson and ethnographic observation of acute care settings. Hence, this paper focuses on findings generated from interviews conducted with patients and their PDM.

### Sampling

Sampling of participants was guided by the Interpretive-Systemic Framework [30, 31], which seeks the perspectives of a wide range of stakeholders engaged with the programme. In accordance with this framework, patients and PDMs were sought on the basis of their engagement with the intervention and which hospital they receive care from.

Respondents were purposively sampled by onsite Principal Investigators attached to 7 tertiary level hospitals, including: Changi General Hospital, Tan Tock Seng Hospital, KK Women and Children’s Hospital, National Heart Centre, Khoo Teck Phuat Hospital, National University Hospital and Singapore General Hospital. These hospitals were representative of acute care settings nationally: there were 8 tertiary level hospitals in Singapore when the data were collected. As the sampling framework had a solid theoretical foundation, the concept of data saturation was not used to guide the number of participants selected for the study.

Onsite Principal Investigators attempted to sample 14 patient-PDM dyads who had completed ACP documentation and an additional 14 dyads who had not. However, owing to difficulties in finding patients and caregivers who had not completed documentation, only twenty-eight participants were interviewed. Thirteen of these respondents identified as PDMs, while the remainder identified as patients.

### Data collection

Collection of data was supervised by PL, a female research fellow who had extensive experience conducting qualitative evaluations of health and social policy interventions in South-East Asia. She collected data with the assistance of OD, WST, and PVP, all of whom were PhD students receiving training in the fields of psychology or health services research. The interviews were conducted in settings deemed comfortable and convenient for participants, which were usually either in their home or in a clinic.

Prior to each individual interview, respondents completed a brief questionnaire recording their demographic details (e.g., age, gender and level of education) as well as the number of ACP conversations they attended. Participants were then interviewed by members of the research team. PL and AH developed two separate interview schedules tailored to reflect how respondents’ role as a patient or PDM influenced their experience of participating in the ‘Living Matters’ programme. Both types of interview schedules were composed of a set of questions and prompts designed to encouraged participants to provide narrative with as little input as possible (e.g., ‘How would you describe your life prior to diagnosis?’). The interview schedules for patients gently guided participants into providing a narrative through focusing firstly on their pathways through treatment, secondly on their memory of the ACP discussion, and thirdly on actions they took after attending the ACP sessions as well as their reflections on their overall experience of the programme. Meanwhile, the interview schedule for PDMs was similar in content but instead focused on their relationship as a caregiver to the patient and their experience of participating in the ACP discussion as a PDM.

The interview schedules were pilot tested during training sessions with members of the research team. All participants only attended one interview, which had an average duration of 45 minutes. Members of the research team developed field notes during the interviews that enabled them to collect data with a reflexive awareness of how their status as academic researchers may impact their relationship with the participants. The research team had not interacted with respondents prior to the interview. Table 1 presents the structure of both interview schedules with sample questions.

**Table 1:** Interview schedule structure.

Dimension of patient interview guide	Sample question	Dimension of PDM interview guide	Sample question
*Pathway through treatment*	"How would you describe your life prior to diagnosis?"	*Patient’s pathway through treatment*	"How did you come to know of your family member’s illness?"
	"How did you come to know of your illness?"		"What did you do to adjust to their illness?"
*Initial ACP session*	"Do you remember your first ACP conversation?"	*Caregiver role*	"What is your level of knowledge on your family’s member’s illness?"
	"Was your family involved in the initial ACP session?"		"Do you receive any support as a caregiver from other members of your family or friends?"
*Steps taken after initial session*	"What actions did you take after the initial counselling session?"	*Role in the ACP process*	"Have you attended any ACP sessions with your family member?"
	"Has your family been involved in the ACP process?"		"How would you describe your role in the ACP process?"
*Assessment of ACP*	"Do you believe that there are any benefits to having the ACP discussion?"	*Assessment of ACP*	"Do you believe that there are any benefits to having the ACP discussion?"
	"How could the ACP programme be improved for other patients and their families?"		"How could the ACP programme be improved for other patients and their families?"

### Analysis

Audio recordings and transcripts of individual interviews were stored digitally and analysed using QSR NVivo [32]. Transcripts were checked for errors by members of the research team, rather than by participants. Themes emanating from transcripts were identified through a framework analysis approach, in which data is usually reduced through a matrix comparing categories of data or cases [33]. Data was analysed according to phases of analysis identified by Pope et al. [34], which started with the research team familiarising themselves with the transcripts. Following this stage, AH, PL, OD, WST and PVP coded according to themes emerging from the data. They, also, met regularly to discuss the analysis and all emergent themes. Finally, themes and cases were compared through a matrix, which were agreed on by all members of the research team.

Trustworthiness and creditability of qualitative data analysis was ensured through a carefully maintained audit trail kept by members of the evaluative team, who meticulously recorded each phase of analysis through memos. Respondents did not provide feedback on the findings.

### Ethics

Research for this study received approval from the institutional review boards of Nanyang Technological University [Ref: IRB-2016-05-023] and the National Healthcare Group’s Domain Specific Review Board [Ref: 2016/00603], which were both situated in Singapore. Prior to each individual interview, respondents were notified of the purpose of the study, selection criteria, their right to withdraw from the study at any point during the discussion and that their confidentiality and anonymity would be maintained. All participants signed an informed consent form prior to commencing the interview, and received a small monetary incentive as a token of appreciation for their time.

During the interviews, only members of the research team and participants were present. Details which could possibly reveal respondents’ identity have been anonymised, with each participant being assigned a code indicating whether they were a PDM or patient. The assignation of PAT denoted that the participant was a patient; PDM was shorthand for Proxy Decision Maker.

## Results

Table 2 displays participants’ demographic characteristics. Participants had a median age of 55 years old (range: 24–84) and the vast majority were female (68%), married (75%) and of Chinese ethnicity (82%). Most respondents identified as Christian (39%), with the remaining identifying as Buddhist (32%), Muslim (14%) or as having no religious affinity (14%). Participants displayed a high level of education, with around half of the sample reporting having attended university (53%).

**Table 2:** Participants’ demographic characteristics (N = 24).

Characteristics	N(%)	Characteristics	N(%)
*Gender*		*Ethnicity*	
Male	9(32)	Chinese	23 (82)
Female	19(68)	Malay	2 (7)
*Type of participant*		Other	3 (11)
Patient	15 (54)	*Marital status*	
PDM	13 (46)	Never Married	5 (18)
*Age*		Married	21 (75)
20–29	1 (4)	Separated	1 (4)
30–39	3 (11)	Divorced	1 (4)
40–49	5 (18)	*Religion*	
50–59	7 (25)	Buddhist	9 (32)
60–69	5 (18)	Christian	11 (39)
70 and over	7 (25)	Muslim	4 (14)
*Type of illness*		Other	4 (14)
Heart disease	12 (43)	*Number of ACP discussions attended*	
Cancer	9 (32)	None	4 (14)
Diabetes	3 (11)	1	22 (79)
Other	5 (18)	2	2 (7)
Multiple comorbities	3 (11)	*Time engaged in ACP programme*	
*Education*		<1 yr	19 (79)
None	1 (4)	1–2 years	2 (7)
Primary	3 (11)	>2 years	4 (14)
Secondary	9 (32)		
Higher	15 (54)		

Almost half of the sample reported that they were either experiencing or caring for a patient with heart disease (43%); while close to a third were dealing with cancer (32%). The remainder of respondents were experiencing or caring for patients with diabetes (11%) or other illnesses (18%), such as osteoporosis. Finally, eleven percent of participants reported that they were dealing with multiple co-morbidities, e.g. heart disease and diabetes.

Majority of participants had attended at least one ACP conversation (86%) within which they completed the necessary documentation. Around three quarters of these respondents (74%) had engaged in the programme less than a year prior to data collection. Only 4 participants had started but not completed an ACP conversation. These respondents had not yet conducted the conversation with their designated PDM and, thus, were unable to complete the documentation.

Table 3 outlines the four major themes with relevant subthemes emerging from participants’ narratives. The first theme was **Engagement with Death** that encompassed factors influencing respondents’ active participation in EoL decision-making processes. This theme was influenced by a set of individual experiential, inter-relational and environmental drivers for their initial engagement with ACP. Individual experiential drivers were related to experiences and belief systems pertaining only to the individual; whereas, inter-relational drivers encompassed decisions influenced by concerns for those close to the participants (e.g. relatives). Environmental drivers comprised of wider socio-cultural norms influencing individual’s attitudes towards death as well as referral processes.

**Table 3:** Themes and subthemes emerging from individual interviews.

Themes	Sub-themes	Description
Engagement with Death	Individual experiential drivers	Personal beliefs
		Perceived need
		Personality traits
	Inter-relational drivers	Witnessing a loved one’s demise
	Environmental drivers	Death Taboos
		Referral by staff
Formation of Preferences	Personal Health Concerns	Perception of quality of life
	Familial Care Concerns	Avoidance of burden
		Discussion of treatment preferences
Choice of Proxy Decision Maker	Values Alignment	Alignment of patient and PDM’s personal belief systems
		Discord between the patient and NHS’s personal belief systems
	Personality traits	Personality traits enabling fulfilment of preferences
	Proximity	Availability
		Emotional closeness
Legacy Solidification	Financial Security	Alleviate out of pocket expenses of EoL care
	Competing needs of other relatives	Consideration of needs of family members after they die

The second theme, **Formation of Preferences,** encompassed concerns influencing respondents’ choice of care, some of which were related to their personal health and familial care concerns. The third theme, **Choice of Proxy Decision Maker,** covered aspects respondents take into consideration when choosing a PDM. Finally, **Legacy Solidification** considers how ACP is used to ensure the welfare of the family after the patient passes.

### Engagement with death

#### Individual experiential drivers

Respondents were driven towards engagement with the ‘Living Matters’ program by their *personal beliefs* related to self-autonomy and control. These personal beliefs held implicit assumptions about the nature of mortality; the underlying concept being that death is a ‘natural’ and unavoidable part of life.

“*The day you are born is the day you start dying*. *That’s how I view things you see*. *So*, *to me*, *death is not something you have to be afraid of because you already start when you are born”*.

PDM11, 61 years old, Female

Although death was perceived as inevitable, it was understood that the actual time point and circumstances under which one could die remained unpredictable. The circumstances demarcated the quality of an individuals’ death, with a peaceful manner of dying being perceived as swift, relatively painless and hindered by few medical interventions, and an undignified death being perceived as painful and prolonged through unnecessary medical treatments that substantially lowered a patients’ quality of life.

Respondents placed importance on ensuring that they died in a peaceful manner, not only to safeguard their own wellbeing, but also to ensure the welfare of those who are emotionally close to them, such as their caregivers, friends and relatives. Under the rubric of their personal belief system the onus is on the patient to set out the conditions necessary for a peaceful death. This required a pragmatic acceptance of death when all options of treatment have been exhausted, which consequently led respondents to prepare for the final days of life:

“*For me*, *you have to be prepared for all this… you don’t know when God will take you*… *I have to do something to prepare for myself*. *I prepare something for my husband to know that what I want if in case at the end of the day I cannot talk or whatever*… *so that they know what you want*.*”*

PAT12, 50 years old, Female

Another individual experiential driver was their *perceived need* to undertake ACP. A few respondents reported that they were more receptive to the ACP programme after being hospitalised for their condition. This type of experience highlighted to participants that their lives could end earlier than they believed it would; thus, bringing an urgency to the need to organise their lives prior to dying, which may include communicating their treatment preferences to others.

“*The start of this year I had septic shock twice… it was quite serious*. *So*, *after I survived those two episodes*, *I kind of*, *realised that I really needed to carpe diem*, *like get as much done as possible… strike out stuff on my bucket list*.*”*

PAT1, 24 years old, Female

A small number of respondents argued that their personal belief system enabled them to develop a set of *personality traits* that made them more receptive towards the ACP programme than other members of the public, who they believed were unduly influenced by death taboos. These personality traits were ascribed as being ‘open-minded’, independent and ‘positive’. Respondents argued their open-mindedness was evident during their social interactions, wherein they displayed an eagerness to discuss topics related to death; while, their independence was exhibited by their active engagement with their treatment. Finally, the trait that they described as positive was viewed as the ability to maintain an optimistic outlook even when experiencing life threatening illnesses:

“*I’m quite open*. *And I take it very positively… I think it’s a good thing*. . .*for me to decide certain things on my own*. *Better than letting my dear partner to decide for me*.*”*.

PAT3, 49 years old, female

#### Inter-relational drivers

Many respondents strongly endorsed concepts underlying ACP, such as patient autonomy and self-determination, after *witnessing a loved one’s demise*. These respondents observed relatives prolonged suffering and lack of mobility, leading them to question the fruitfulness of sustaining their lives. Observing others suffer often shaped participants’ personal beliefs concerning death:

“*You know sometimes when I see the way my sister suffer (from cancer) right*?.., *Of course you pray and then you ask that she recover*, *but towards the last was very terrible for her*. *It was terrible pain*, *very agonizing pain*. . . . *I don’t think I would like to go through something like that or see somebody go through like that*. *And sometimes you think you know it should end*.*”*

PDM11, 61 years old, female

“*Unless they (members of the public) see it themselves*. *I mean frankly speaking–they see somebody suffer*. *I think they will consider signing it (ACP)”*.

PDM13, 62 years old, Female

#### Environmental drivers

The primary environmental driver for engagement with the ACP programme was *referral by staff*, as only three participants had actively sought out a conversation after learning of the program through a relative or engagement with promotional activities. For instance, PAT6 requested an ACP session after learning about the existence of the programme through his nephew, who had previously worked at one of the local hospitals. These findings indicated there remained a lack of awareness of ACP in the general public.

Remainder of respondents reported that they were informed of the programme and then later referred to an ACP session by a member of staff at a hospital they were attending. Many of these participants were referred to the ACP programme at a fairly late stage of their illness- there were two cases of referral for a session conducted only with the PDM due to difficulties in communication or the patients not being deemed as cognitively able enough to choose their own treatment preferences. In one such dyad, the PDM attempted to protect her father from stress induced from discussion of topics related to death through forbidding the ACP coordinator from talking to him, which ultimately resulted in him being excluded from future ACP discussions:

“*He (The ACP Coordinator) just go direct and say*, *‘Why you want (ACP) there*?*’*. *My dad get angry*. *(His wife) called me and said she didn’t talked to my father this time*. *So that’s why I say*, *‘No*, *you shouldn’t directly ask my father this kind of sensitive questions… Because he get upset*. *He’s high blood shoot up on that day…*. *Very high*. *He got worried*. *And so I say*, *‘You know*, *anything*, *just talk to the family*.*’ Actually he can’t talk to the patient direct*. *Because I think the family can just help them to decide*. *Okay*.*”*

PDM5, 49 years old, female

In other cases, respondents were introduced to the ACP programme by staff, who they had already developed an emotional bond with. A distinguishing feature of these participants was that they had acquired a social network consisting of other patients and HCPs within their acute care setting and were to some extent involved in an informal type of activism. For instance, PAT11 had set up a workshop on ACP for her patient support group. As medical staff already knew these participants well, they were able to discuss fairly personal topics without fear of reproach. One patient stated:

“*For me*, *it’s because I’ve become very close with them (medical staff)*. *So they know the nurse approach the proper person so that they brought it up*. *Because for me*, *anything will do*. *So that’s the time when she (the nurse) asked me–she was very careful*. *She slowly… explained (ACP) and says*, *‘It’s nothing*. *Just think about it’*.*”*

PAT5, 61 years old, female

Participants did, nevertheless, contend that the continual existence of pervasive *death taboos* made elderly Singaporeans resistant to concepts underlying ACP as they believed that any mention of death could hasten their demise. Some participants purported themselves to be impervious to such taboos due to their minority religious status either as a Christian or Muslim, which enabled them to be more accepting of death due to their belief in the existence of an afterlife.

“*Asian culture (death) is very taboo*. *But I can swear to you erm… the 50 something lot or the 60 something lot will have no problems… deciding and so on*.*”*

PDM2, 54 years old, Female

There were a small number of respondents who used concepts underlying filial piety to justify their endorsement of ACP. These participants would argue that the theoretical foundation of filial piety is for younger relatives to fulfil the wishes of elder family members whilst they are alive and well rather than forcing them to undergo painful and unnecessary treatment to prolong their lives:

“*In Cantonese*, *there is a saying*, *‘…you are filial when they are alive*, *after they die*, *you don’t need to be filial’”*

PAT9, 84 years old, female

“*So I don’t want you all to have that burden where*, *you know*, *the Chinese have the saying*, *‘Where you let go*, *people comment*. *You don’t let go*, *you all will suffer’*. *So I said*, *‘No*, *you don’t have to decide anything at all*. *I decide myself*.*’ That’s all*.*”*

PAT6, 66 years old, male

The aforementioned quotes demonstrated that it is possible to use traditional social norms in South-East Asian cultures to affirm, rather than reject, concepts underlying ACP, such as quality of life. These concepts were endorsed from a collectivist standpoint- participants argued that filial piety can only be fulfilled through abiding by parents’ or elder relatives’ treatment preferences instead of prolonging their suffering through unnecessary treatment. Respondents’ reinterpretation of filial piety could, thus, highlight potential new avenues for the development of culturally appropriate public health campaigns for ACP.

### Formation of preferences

#### Personal health concerns

Personal health perception, particularly those concerning quality of life, influenced respondents’ formation of preferences. Their *perception of quality of life* was defined as having a minimal level of discomfort and the ability to independently conduct their daily activities. The level of discomfort that they were able to endure were demarcated by their past experiences of pain and illness. This resulted in respondents expressing a wide spectrum of preferences, which spanned from being fairly minimal (i.e. the need to feel ‘clean’) to covering risky medical procedures:

“*I’m saying no to that (tracheostomy)*, *but I’m okay with intubation… Because*, *I don’t know*. *It (a tracheostomy) seems really scary… I think it’s just weird to have a hole there…you can see the scar is quite obvious… But intubation*, *you know*, *can put in a tube and then take it out and then no one would be the wiser*.*”*

PAT1, 24 years old, female

“*Let’s say–okay*, *let’s say you’re bed ridden*. *I say ‘…(at) least must clean me up la*. *I don’t care what you do*. *You want to poke me–I’m fine*. *I need to be clean*, *you know*, *shower*.*’”*

PAT11, 36 years old, female

#### Familial care concerns

Meanwhile, formation of preferences was shaped by patients’ and PDMs’ concerns for the psychological welfare of those closest to them. These participants were concerned that their loved ones may carry the psychological burden of witnessing their unnecessarily prolonged and painful death, as they had to for their deceased loved ones:

“*We (patients) don’t want to be a burden to them (children)*. *I mean why prolong*? *For me*, *I always believe in quality life*. *I want quality life*. *I don’t want quantity*. *Don’t need for that”*.

PAT14, 71 years old, Female

Responsibility for decisions on end-of-life care was framed as an unnecessary psychological “burden” as family members are obligated to make significant decisions on treatment, such as whether to discontinue life support when the patient is in a vegetative state, with little knowledge of what their loved one would have wanted. Participants contended that such decisions could cause distress for family members who are worried their choices could hurt those closest to them. These types of decisions could potentially cause conflict between family members, who may have different perspectives on what types of treatment to follow. The following quotes underscore how treatment preferences were formed with the purpose of alleviating psychological “burden” for family members:

“*There are many cases that when something happen*, *the families are in conflict sometimes*. *Like this person wants this*, *this person wants this*, *you know*? *That’s why conflict*, *like uh… the immediate family and the relatives you know*? *There’s so much conflict*. *So if you put in writing you know what the patient wants*. *Because during that period*, *he cannot decide”*.

PDM6, 54 years old, female

“*I would say that it’s good*. *Especially if we have*, *ever since family in intensive care unit or neurosurgery ward*. *Why it’s good is because there is a guideline given to the family*.*”*

PAT2, 44 years old, male

Few participants actively engaged in behaviours to inform their preferences; only three mentioned that they ‘researched’ their treatment options, which usually involved a search on an internet database. Formation of preferences did not seem to be influenced through *discussion of treatment preferences* with relatives, although their welfare formed a substantial part of patients’ decision-making processes. Instead, patients often chose to inform family members of their preferences once they had settled on them. PAT10 described her discussion of preferences with her PDM, who is her niece, when she said my “niece discussed–I explained to her and she listened, ‘in the future, if there are any changes that I can’t walk or what, I don’t want to count on you’”. Similarly to other patients’ accounts of their discussion of preferences with family members, it is apparent that the conversation was conducted in a linear fashion with the intent of seeking endorsement from those who could be later responsible for their treatment.

### Choice of proxy decision maker

#### Values alignment

Decision-making processes behind choice of PDM were influenced by perceived alignment of personal belief systems between both parties. Some participants stated that a PDM was chosen on the basis that they held the same attitudes towards death as the patient did. For instance, PDM11 chose her niece as a spokesperson due to their shared belief in Christianity and their lack of adherence to death taboos:

“*We share the same faith*, *number 1*. *Number 2*, *her outlook on things also not very…for one of the better word*, *traditional…*.*I don’t think anybody thinks like me because… I tell people*, *‘the day you are born you are on the road to die’*, *and they will be like*, *‘how can you say something like that*?*’*. *You know what I mean or not*? *I don’t know why they are so shocked because logically if you look at it*, *it’s like that… My niece… (is) very strong in her faith so I think it’s very easy… (she is) very firm also*, *anybody argue*, *any other way*. *She will tell them what she dislikes and that’s it*.*”*

PDM11, 61 years old, female

PDM11’s account of factors motivating her choice of spokesperson also points to perceived personality traits as she argues that her niece firm resolve and honestly imbues her with the ability to execute difficult decisions. A few other participants cited personality traits as a motivating factor in their choice of spokesperson. As with PDM11, these participants believed that their chosen spokesperson held a set of personality traits that imparted them with the ability to fulfil their preferences. For instance, PDM7 argued that she was chosen as a nominated health spokesperson by her aunt as she was ‘strong’ in contrast to her uncle (the patients’ husband) who was deemed as too ‘soft’ and ‘sensitive’ to ‘unplug’. She noted that other family members would be “too sad to even think or do anything to fulfil her command (preferences)”; perhaps, demonstrating that she believed that they lacked stoicism in the face of adversity.

There were, nonetheless, some participants who were unable to find a PDM who held a similar personal belief system to themselves. In a few cases, discord between the patient and PDM’s personal belief systems had little influence on implementation of the programme as the proxy decision maker chose to endorse patients’ wishes. For example, the dyad of PDM12 and PAT1 held contrasting religious beliefs, with one describing herself as a Christian, while the other was a self-ascribed ‘free-thinker’. Due to their contrasting personal belief systems they disagreed on treatment preferences- PDM12 preferred to immediately discontinue life support which clashed with PAT1’s wish to wait for 2 weeks. Despite these differences in opinion; PDM12 strongly endorsed her decisions, stating, ‘I think she herself know her conditions’.

Other patients did perceive discord between the patient and PDM’s personal belief systems as a potential barrier to fulfilment of their preferences. These participants reported that their chosen PDM were reluctant to fully participate in the ACP session due to difficulties in the relationship or long held death taboos. For example, PAT2 did not conduct the ACP conversation with his wife as they were experiencing marital discord; while, PAT4 reported that during the ACP discussion his wife displayed a reluctance to partake in the conversation, asking, ‘Must we really do this?’. He believed that the ACP conversation frightened his wife and reported that since the session:

‘*I just don’t want her to be reminded of it again*. *Cause like I say…she still have traditional view of death*. *So speaking about death is not so comfortable to her*.*’*

PAT4, 55 years old, male

This quote demonstrates that PAT4 avoids discussion of ACP or even topics related to death in order to protect his wife from potential psychological trauma. Meanwhile, PAT2 expressed the concern that “I will worry if my wife respect what I want. By the time I won’t know already”. These particular cases indicate that some PDMs may need additional psychological support to cope with their own death anxiety or negotiate difficulties within their relationships to the patient.

#### Proximity

A few PDMs were chosen on the basis of their *availability* to partake in the entirety of the ACP programme from initiation of the conversation to enactment of wishes. These PDMs were chosen in place of other relatives who were often not available to partake in the ACP process due to familial disputes or other commitments. PAT7 chose her close friend, PDM4, as her proxy decision maker as her relationship with her son had been fraught since divorcing her husband. Meanwhile, PAT10 chose her niece, PDM3, as proxy decision maker as her siblings had overriding family commitments:

“*All my brothers are not free*. *They have their own family and work to take care of*, *I cannot count on them*. *Because I am staying with my niece–because I told my fourth brother*, *my fourth brother told me that it is good that I am staying with my niece*.*”*

PAT10, 50 years old, female

In other cases, proxy decision makers were chosen on the basis of their *emotional closeness* to the patient. Their emotional closeness was considered as a product of them being related as a member of the family or partner, which often imbued them with knowledge of patients’ character and belief systems. As PAT6 stated, ‘I think no matter how–your partner is usually the person who should understand you more’. It was, also, forged through shared experiences and prolonged proximity. These shared experiences were described in terms of provision of mutual care and support. PDM7 was chosen as a proxy decision maker by PAT12 as she frequently supported female members of her family in deciphering financial documents, which may have given her the necessary skills to navigate the semi-legalistic ACP process. PDM11 developed a close emotional bond with her niece through caring for her:

“*I have already been making a lot of decisions or helping to make the decisions that she needs when she’s alive*. *So I*.. *can help with her decisions… And then even now also*, *more or less*, *and ya that’s why I nominated [by] her*.*”*

PDM11, 61 years old, female

A small number of proxy decision makers were chosen on the basis of their emotional closeness to the patients rather than on alignment of values. As noted earlier, this method of selecting a PDM sometimes resulted in tensions between both parties, which was apparent through proxy decision makers’ reluctance to actively partake in the ACP conversation. Patients chose these PDMs despite concerns that they may not execute their treatment preferences as they were keen to maintain their status within the family:

“*It take two hands to clap*. *I can only tell the boss that there’s a weaver there*. *I cannot put the weaver into the water*. *Correct*?*”*

PAT2, 44 years old, Male

### Legacy solidification

Both patients and PDMs used ACP with the purpose of securing their enduring legacy after death, which was symbolised through the need to stabilise the family structure. Some participants’ formed preferences in the hope that it would alleviate the financial burden of out-of-pocket expenses related to EoL treatment for relatives. Patients, in particular, displayed a keen awareness of the competing needs of other relatives; arguing that these family members would still need to pay for living costs after they pass away. These patients believed that if there was no possibility that they could survive their treatment, they would be incurring an unnecessary financial burden on those who outlive them:

“*Every one of them needs to work*. *Now*, *if the younger generation does not have jobs*, *cannot*. *Need to pay for the house*, *need to pay for the children*, *right*? *And you are lying there doing nothing everyday*, *need to go is more scary*. *Never mind*, *I have thought this through*, *need to go*, *then have to go*, *to Heaven is ok*. *When you are old*, *there is nothing much*, *don’t burden the younger generation*.*”*

PAT9, 84 years old, female

## Discussion

This paper makes a unique contribution to the literature on ACP in South-East Asian countries by examining patients and caregivers’ decision-making processes at each phase of a national-level intervention in Singapore [28]. The findings of this paper differed from those of other studies on ACP and/or Advance Directives that were conducted with ethnically Chinese populations on two fronts. First, these studies identified sociocultural norms associated with pervasive death taboos and filial piety as acting as a barrier to participation in a given intervention [35, 36]. Although participants in this study were keenly aware of these sociocultural barriers, they were also able to point to factors facilitating their acceptance of ACP. One such facilitator was their demographic characteristic of belonging to a minority religious group (Muslim or Christian). These respondents argued that their belief in an afterlife encouraged their involvement in the programme.

Moreover, findings pertaining to **Engagement with Death** revealed that participants’ endorsement of the concepts of self-autonomy and control were influenced by their experiences of witnessing their loved ones suffer towards the end of their lives and their own personal beliefs on death and dying. In combination, these factors enabled a few participants to reinterpret the concept of filial piety [36] to endorse elderly patients’ control over their treatment, arguing that it is the duty of children to follow their parents’ wishes rather than to unnecessarily prolong their lives [26]. These findings indicate that it may be possible to encourage acceptance of ACP among ethnically Chinese populations by proposing an interpretation of filial piety that supports elder patients’ needs and dignity [37].

The second way in which this study contrasted from literature on ACP with Chinese populations was that its patients independently chose their own treatment, with family members playing a supportive role [29, 37]. Findings on **formation of preferences** indicated that participants’ decisions were influenced by concerns about personal health perceptions and familial care. The former concern was formed through patients lived experiences of their illness, which often shaped their perception of the level of pain and disability they were able to tolerate towards the end of their life. Meanwhile, the latter concern revolved around alleviation of psychological ‘burden’ incurred by responsibility of caregivers for EoL care.

Moreover, results on the **Choice of Proxy Decision Maker** demonstrated that some patients chose a nominated health spokesperson based on their religion and their personality traits, such as stoicism. There were, however, other respondents who were unable to secure a PDM who shared their particular personal belief system owing to familial discord or traditional values around death. In a few of these cases, patients chose PDMs emotionally close to them in order to enable **Legacy Solidification** by maintaining the family unit. Although a few of these patients were anxious that their preferences may not be adhered to, they seemed to be willing to sacrifice their autonomy to ensure long-term stability for their relatives and loved ones.

These findings indicate that the ‘Living Matters’ ACP programme may not cater to the needs of patients who are unable to find nominated health spokespersons who fully endorse their treatment preferences. As of 21^st^ September 2018, the Professional Deputies and Donees scheme was launched, allowing for individuals to hire a paid professional donee to make decisions on their behalf should they lose their mental capacities. This scheme should enable patients to find proxy decision makers who adhere to their documented preferences. It does not, however, cater to patients, whose overriding concern is to maintain their family unit through making one of their loved ones a proxy decision maker to signal their emotional closeness, highlighting a need for extra psychological support for PDMs with anxieties surrounding death.

**Fig 1:**
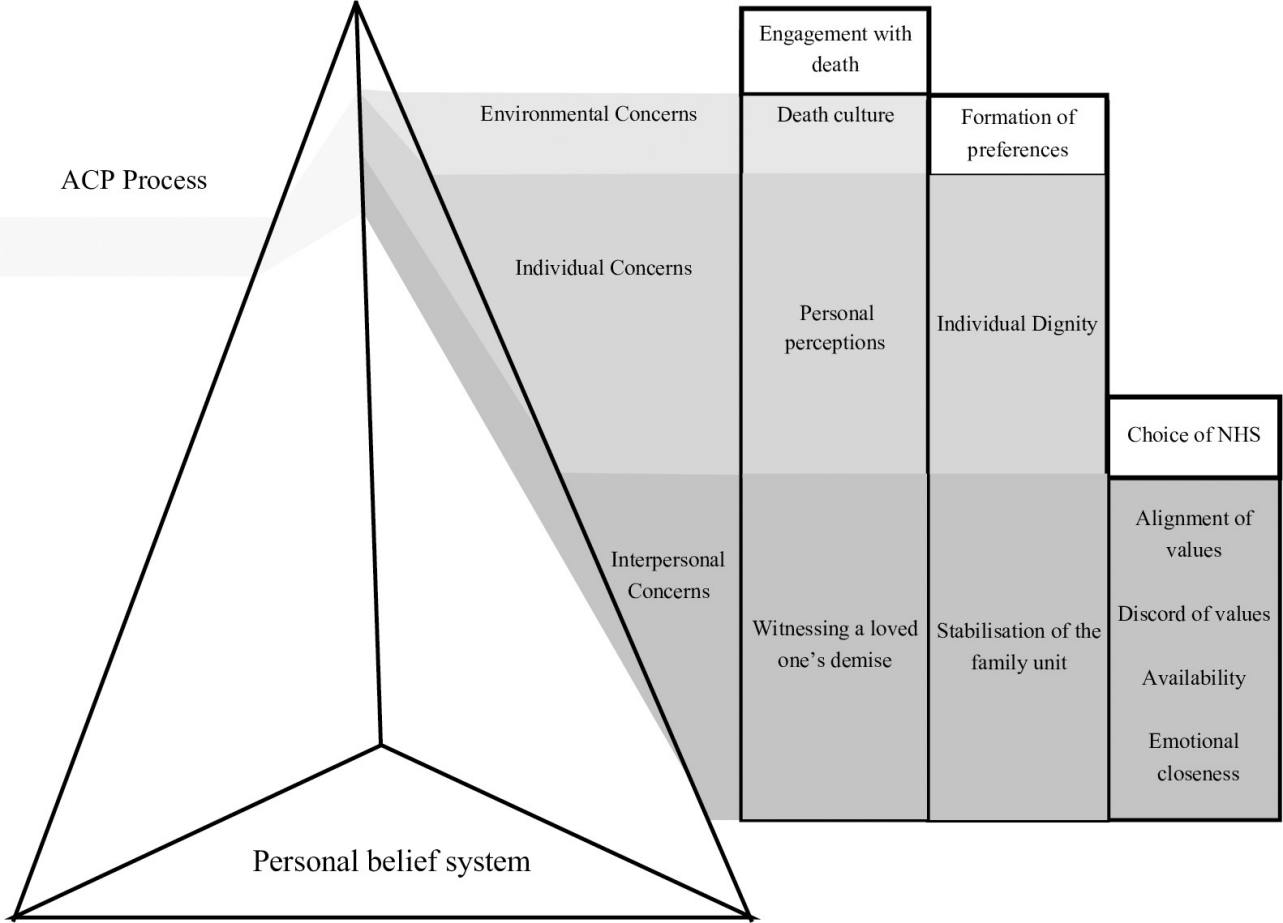
Directive decision-making process framework.

The overall findings of this study have led to the development of the directive decision-making process framework (displayed in Fig 1). This framework delineates personal and sociocultural factors influencing participants’ decision-making processes. Respondents’ continual participation in the intervention is driven by their personal belief system, which acts as a prism through which they interpret religious doctrine and socio-cultural norms according to their own needs. The prism of their personal belief system refracts in light of the ACP process to reveal a set of environmental, individual, and interpersonal concerns at each phase of interaction with the programme. Environmental concerns refer to influences associated with one’s social environment, while individual concerns focus on their self-perception and lived experiences. Finally, interpersonal concerns encompass their relationship to others close to them.

The directive decision-making process framework indicates how the Respecting Choices’ model [11, 12] can be adapted to the Singaporean context through an appreciation of how individuals’ personal belief systems can influence their decisions at each phase of the ACP process. Respondents’ initial participation with the programme, or engagement with death, is influenced by environmental, individual, and interpersonal concerns. Individual concerns focus on personal perceptions of death and dying, while interpersonal concerns highlight how witnessing a loved one’s demise may ensure that patients are more receptive to the philosophical foundations of ACP. Environmental concerns refer to death culture in Singapore. As this seems to be the only phase of the intervention affected by environmental concerns, this framework suggests that it may be possible to encourage receptivity to ACP through a culturally sensitive public health programme that promotes acceptance of death and dying [38].

The next phase of the framework, **Formation of Preferences**, was influenced by a combination of patients’ individual and interpersonal concerns. Their individual concerns encompassed their individual dignity, which referred to their sense of personhood and self-worth toward the end of their life [39]; their interpersonal concerns focused on stabilisation of the family unit after passing. The final phase is dominated by interpersonal concerns encompassing alignment of values, discord of values, availability and emotional closeness with one’s PDM. These interpersonal concerns indicate that personal attitudes towards death and dying play as much of a role in patients’ choice of PDM as their perception of the relationship with this individual.

In summary, the directive decision-making process framework highlights an underlying weakness in the conceptual framework of ACP, which holds individual autonomy as central to decision making on EoL care. Current research suggests that the concept of individual autonomy can be incongruent with the concept of autonomy in Asian cultures, in which families collectively make decisions [28, 40, 41]. This study highlighted that even when patients are directing the decision-making process, they often do not act as autonomous agents who are untethered to their social context and solely focused on their own wellbeing [24]. Instead, their decisions are often dominated by familial care concerns, as they attempt to solidify the family unit after their eventual death. This finding concurs with that of other studies in Eastern and Western countries, which suggested that patients often make treatment decisions with their loved ones in mind [42–44]. Hence, this study highlights that the conceptual framework of ACP may need to be adapted to balance patients’ need for autonomy with their wishes to ensure the wellbeing of those they leave behind.

### Limitations

This is one of the few studies to seek the perspectives of patients and proxy decision makers who had differing levels of engagement with an ACP programme from a wide array of acute care settings. It, nonetheless, had its limitations, namely, that it was not possible to sample the originally envisioned number of participants owing to difficulties recruiting respondents who had not completed documentation related to the conversation. This was due to records not being kept on patients or PDMs who had not completed the documentation within acute care settings. There were, also a number of patients and carers who were reluctant to undertake the interview due to the controversial nature of the study topic. Hence, it is possible that the research team were only able to sample participants who were already highly motivated and willing to speak about death and dying.

## Conclusion

This study reveals that many participants made decisions on their EoL care based on their perceived long-term legacy, which resulted in the **Formation of Preferences** and **Choice of Proxy Decision Maker** being used to ensure that patients’ family units remain with a solid financial and psychological foundation after their death. These findings point to possible new avenues for research and policy. In terms of research, difficulties with recruitment of participants for this study highlighted the need for further qualitative and quantitative research on factors influencing lack of receptivity to ACP in South-East Asian countries. Regarding policy, this paper indicated that the ‘Living Matters’ ACP programme needed to offer death education and psychological support to potential proxy decision makers, especially in cases where they are reluctant to approach topics related to death and dying.

## Supporting information

S1 Table COREQ (COnsolidated criteria for REporting Qualitative research) checklist. (DOCX)
